# A three phase optimization method for precopy based VM live migration

**DOI:** 10.1186/s40064-016-2642-2

**Published:** 2016-07-08

**Authors:** Sangeeta Sharma, Meenu Chawla

**Affiliations:** Department of Computer Science and Engineering, Maulana Azad National Institute of Technology, Bhopal, 462003 India

**Keywords:** Virtualization, VM live migration, Service interruption, System management, Total migration time, Downtime

## Abstract

Virtual machine live migration is a method of moving virtual machine across hosts within a virtualized datacenter. It provides significant benefits for administrator to manage datacenter efficiently. It reduces service interruption by transferring the virtual machine without stopping at source. Transfer of large number of virtual machine memory pages results in long migration time as well as downtime, which also affects the overall system performance. This situation becomes unbearable when migration takes place over slower network or a long distance migration within a cloud. In this paper, precopy based virtual machine live migration method is thoroughly analyzed to trace out the issues responsible for its performance drops. In order to address these issues, this paper proposes three phase optimization (TPO) method. It works in three phases as follows: (i) reduce the transfer of memory pages in first phase, (ii) reduce the transfer of duplicate pages by classifying frequently and non-frequently updated pages, and (iii) reduce the data sent in last iteration of migration by applying the simple RLE compression technique. As a result, each phase significantly reduces total pages transferred, total migration time and downtime respectively. The proposed TPO method is evaluated using different representative workloads on a Xen virtualized environment. Experimental results show that TPO method reduces total pages transferred by 71 %, total migration time by 70 %, downtime by 3 % for higher workload, and it does not impose significant overhead as compared to traditional precopy method. Comparison of TPO method with other methods is also done for supporting and showing its effectiveness. TPO method and precopy methods are also tested at different number of iterations. The TPO method gives better performance even with less number of iterations.

## Background

Cloud Computing is considered as an utility based system for the dynamic provisioning of IT resources and services. For providing on-demand and flexible provisioning of resources and services, it needs to utilize resources efficiently without any interruption due to maintenance and setup issues. For this purpose, resources are shared among various users in such a way that the requirement of all users can be fulfilled. Virtualization makes it possible by running multiple operating systems and multiple applications on a single physical machine. It divides physical machine into two or more virtual machines and each virtual machine operates as an independent environment. By means of virtual machine, cloud computing fulfills demands of resources and services for multiple users simultaneously. For better performance, simply assignment of resources is not sufficient, but an efficient utilization of resources (Mishra et al. 2012) is equally important. Virtualization gives facility of managing virtual machines in such a way that it works without any service interruption. Virtual machine live migration is one of the key features of virtualization, which moves virtual machine from one physical machine to another machine, without or least service interruption.

**Fig. 1:**
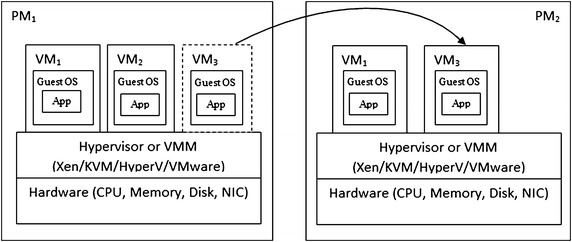
Virtual machine migration in virtualized physical machine

With the help of virtual machine live migration, cloud administrator utilizes resources more efficiently by balancing load between under and over utilized servers without interrupting virtual machine services. Virtual machine live migration also helps to reduce power consumption in virtualized datacenters by moving virtual machine from underutilized servers to other servers and shutting down the idle servers. Because of these benefits, virtual machine live migration is widely being used by datacenter administrators, as a result, a huge number of virtual machine migrations are taking place. Migration of a huge number of virtual machines consume a large amount of network bandwidth as well as imposes performance overhead, which adversely affects the overall efficiency of the system. In order to address this issue, it is required to use efficient virtual machine migration method which imposes minimum overhead. Figure 1 represents the architecture of virtual machine live migration in which virtual machine is transferred from one physical host to another.

Hypervisor or Virtual Machine Monitor is a tool or a program which is used to implement virtualization. It acts as an interface between underlying hardware and the virtual machine. It performs all virtual machine management tasks without interrupting virtual machine services. Various commercial and open source virtualization tools are available. *Xen* is one of the open source virtualization platforms which is widely adopted by research communities due to its availability and ease of adaptation. Other than Xen, *KVM* is another popular open source virtualization platform. VMware of *VMware Incorporation* and HyperV of *Microsoft Corporation* are some other widely used commercial virtualization platforms.

Xen supports two types of virtual machine live migration methods, one is precopy as default method and another is postcopy which is used as an alternative. Precopy method is widely used for virtual machine migration as compared to its counterpart namely postcopy. This is because postcopy method is prone to destination side crash from which recovery is not possible. Basic components of virtual machine which are required to be transferred in the migration process are CPU state, device state, storage state, and memory state. In the present scenario, most of the cloud based data centers are equipped with network attached storage (NAS), and data is accessible by all hosts. Taking advantage of network attached storage, only internal state of CPU and device with consistent memory state are transferred in virtual machine migration. The CPU and device state data is negligible as compared to the memory state data, so main focus is on efficient migration of virtual machine memory. Precopy method iteratively transfers updated memory pages to migrate consistent image of virtual machine. As a result, redundant data is transferred several times which leads to increase in number of pages transferred and hence, increases the migration time. Precopy method moves virtual machine across hosts with less service interruption, but there is still scope for improvement. In order to reduce overhead imposed by migration methods, it is required to make virtual machine migration method efficient. This paper presents an efficient three phase optimization (TPO) method for precopy based virtual machine migration with low overhead.

The main objective of this paper is to show the requirements of an efficient virtual machine migration method, analyze the effect of virtual machine migration method on performance parameters, and develop an efficient method for virtual machine migration for virtualized data centers, so that virtualization can manage virtual machines efficiently and facilitate cloud computing as an effective and useful technology. Specifically, this paper aims to:

- Investigate basic virtual machine migration precopy method that moves virtual machine from one host to another with less service delay.
- Develop optimized method for precopy based virtual machine migration in order to manage virtual machine efficiently with minimum service interruption and overhead.
- Study impact of the work by analyzing the experimental results on standard benchmark workloads and evaluate its performance based on experimental results.

Rest of the paper is organized as follows:

Section “Related work” describes the related work. Section “Background knowledge” provides the framework of basic virtual machine migration precopy method. Section “Three phase optimization (TPO) method” presents the proposed TPO method for virtual machine migration. Section “Simulation and performance evaluation” shows the performance analysis of proposed TPO method. Section “Open issues” discusses the vision on open issues in virtual machine live migration. Section “Conclusion and future work” concludes the paper with future research directions.

## Related work

This section explores existing works related to improvement of precopy method.

Waldspurger (2002) introduces several virtual machine memory management mechanisms and policies for VMware ESX server as a virtualization platform namely memory ballooning, content-based page sharing and hot I/O page remapping. In memory ballooning, physical host fulfills excess memory demands of certain virtual machine by retrieving unused memory from other virtual machines. In content-based page sharing and hot I/O page remapping, it shares those pages between virtual machines which are having same content and frequently used pages by I/O events. These techniques are used individually or collectively to support the feature, memory over commitment of hypervisor, that allows virtual machines to demand or use memory space more than the available in physical host.

Nelson et al. (2005) shows design to provide fast transparent virtual machine migration and uses Vmotion, an integral part of VMware product for implementation. It gives downtime as a performance measurement for hundreds of concurrently running virtual machines with standard industry benchmark workloads. It also shows overheads and resources required during virtual machine migration.

Clark et al. (2005) presents one of the first works which uses precopy method for virtual machine live migration and uses Xen as a virtualization platform. In this it traces writable working set (WWS), which is a set of frequently updated pages and it will be sent at the end, instead of each time they are updated. In this way, it reduces transfer of redundant pages. The paper also introduces another method named ‘dynamic rate limiting’, to further enhance the performance of the virtual machine live migration. This method adapts dynamic bandwidth limit with respect to dirty rate for transferring of pages during migration. Which results into decrease in downtime by increasing bandwidth has been shown.

Ma et al. (2012) presents a compression based approach, in which authors use a compression technique, called as Run Length Encoding (RLE), to reduce number of pages transferred during migration. Reduction in total pages transferred during migration leads to reduction in total migration time and downtime. The RLE technique compresses only allocated pages instead of all pages. For this, it uses Linux memory management mechanism buddy system which scans whole virtual memory and maintains a list of unallocated pages. During migration, in place of sending unallocated page, only one byte NODATA is sent. In this way, it tries to reduce total pages transferred during migration by sending only allocated pages in compressed form, and one byte NODATA for each unallocated page. Memory exploration module used in this work is guest dependent. For different guest operating systems, it requires to write different exploration module. Compression algorithm RLE reduces total data transferred as well as migration time but increases overhead of compression/decompression.

Svärd et al. (2011) also used compression approach with dynamic page transfer reordering. This approach orders the page transfer in such a way that retransfer of frequently updated pages is reduced. Based on the number of times a page is being updated during migration, page weight for each page is calculated and pages are reordered accordingly. This results into reduced number of pages transferred during migration. For further improvement, authors combine this idea with delta compression technique which leads to reduction in both migration time as well as downtime. Delta compression reduces amount of data transferred by sending XOR deltas between previous and current page versions instead of full page. It is highly dependent on fast, efficient page privatization schemes and compression. Here, again overhead is high.

Zhang et al. (2010) presents another method based on compression approach with data deduplication in migration. By utilizing the self-similarity of run-time memory image, authors apply RLE method during migration to remove redundant memory data. For calculating the similarity of pages, hash based fingerprints are used. For this, it maintains two FNHash and FPHash LRU hash tables. This approach improves migration performance with space and CPU resource overhead.

Jin et al. (2009) presents an adaptive memory compression method for migration. It analyses memory data to find regularities within it and divides memory pages in three categories: memory with many zero-bytes, memory with high similarity and memory with low similarity. Based on the category of memory pages, compression algorithm is applied to balance the overhead of compression. This approach tries to improve the performance of migration method while balancing the overhead due to compression. Compression based methods are affected by the overhead occurred due to compression/decompression process and to overcome from this, Jin et al. (2011) presents another method using CPU scheduling. In this method, it controls the memory dirty rate by using CPU scheduling in such a way that dirty rate reaches to an acceptable desired small amount. The idea behind this is, to control the dirty rate as performance of migration method highly depends upon the dirty rate. This improves the performance of migration method specially downtime, which is one of the important performance parameters. Overhead occurred due to CPU-Scheduling affects the application performance of all the guest virtual machines.

Similar to Jin et al. (2011), Liu et al. (2010) presents a slowdown scheduling algorithm. This algorithm reduces the dirty rate by adjusting CPU resources allocated to the migration domain. This results into decreasing CPU activity, and reducing dirty page rate. It improves migration performance but also affects the application performance running within the migration domain.

Liu et al. (2011) presents a fast and transparent migration method for LAN as well as for WAN environment. It uses checkpointing/replay and trace/replay mechanism and generates log files, a record of nondeterministic system events during migration. In place of actual pages, these log files are transferred which are smaller in size, through a synchronized algorithm till source and target have a consistent virtual machine state. In this way, it reduces downtime as well as network bandwidth consumption.

 Liu and Fan (2011a) presents an approach which is based on instruction execution trace/replay mechanism with CPU scheduling. This method also transfers log file in place of complete page and also manages the speed of log file generation by adjusting CPU scheduling. It improves migration performance, but it has not been tested for complex environment.

Jo et al. (2013) uses shared network attached storage between hosts to improve migration. In this method, memory-to-disk mapping is transferred in place of the page itself. Page can be directly fetched from network attached system and for maintaining consistency, P2b (Page to block) map is used. The main downfall of this method is downtime, which increases significantly when the virtual machine is idle because almost all available memory is duplicated on disk. Another drawback is that virtual machine cannot be resumed till the background process is not completed which significantly affects the downtime. This method can only be applied where hosts are connected with shared storage.

 Alamdari and Zamanifar (2012) presents a prediction based approach, named reuse distance. This approach tracks frequently updated pages and keeps them till the last iteration in order to reduce retransfer of same pages. It adopts the concept of reuse distance to track frequently updated pages and based on this, decision of transferring dirty pages in each phase is taken. Reuse distance of a page can be calculated as the number of distinct pages updated between two consecutive updates of same page and for this, it manages the reuse distance bitmap. Based on reuse distance value, those pages that have small reuse-distance are marked as frequently updated pages. In this way, it reduces the transfer of same page iteratively which leads to reduced total number of pages transferred, migration time and downtime as well.

Hu et al. (2011) too presents the prediction based time-series method. It also traces the frequently updated pages by using an array of historical bitmap, which stores past historical statistics (size N) of sent pages. Based on this, the method predicts frequently updated pages and sends them at the last iteration. It also places bound to the number of iterations, by taking optimal ratio of threshold of frequently dirty pages (K) and maximum size of historical bitmap (N). By taking correct value of K, the size of historical bitmap is maintained, as well as number of iterations is also reduced. Ma et al. (2010) also uses an extra bitmap to mark frequently updated pages which are sent in last iteration. Here, maximum number of iterations is kept as five. The result shows reduction in total pages transferred and migration time but with increased downtime.

Liu et al. (2011b) presents two models based on prediction to evaluate the migration cost in terms of performance as well as energy. By using different workloads, it simulates migration process to predict the migration cost. It significantly reduces migration cost along with significant energy saving. In this work, migration cost for both performance and energy is estimated.

The above works discussed, all try to reduce total pages transferred by reducing the total size of transferred pages either by compression, managing dirty rate, keeping frequently updated pages or by transferring log files. Along with total pages transferred, all of them also try to reduce total migration time and downtime. Most of the works are unable to improve all three parameters simultaneously and reduce overhead imposed by the migration method. Here, only precopy based methods have been discussed, Sharma and Chawla (2013) gives the review of both precopy and postcopy based methods.

This paper is also based on predicting frequently updated pages and groups pages into frequently and less frequently updated pages. The proposed TPO method optimizes migration method from very first iteration till last and improves performance parameters effectively with low overhead.

## Background knowledge

This section describes the working of precopy method, performance parameters, the basic architecture of Xen and its virtual machine live migration mechanism to execute precopy method.

**Fig. 2:**
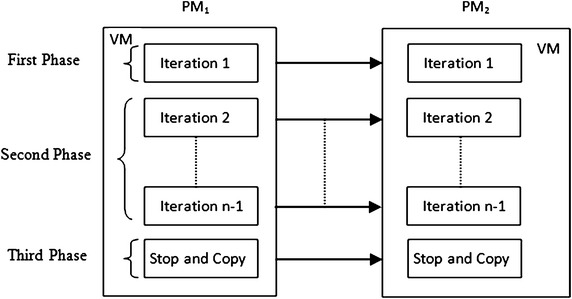
Iterations in precopy method

Virtual machine live migration method effectively manages the transfer of virtual machines and improves the efficiency of virtualized datacenter. Precopy is a widely used method for virtual machine live migration, which is adopted by Xen and VMware. Precopy iteratively transfers virtual machine from source to target host. The basic architecture with iterations performed in precopy method is shown in Fig. 2. It is mainly divided into three phases.

First phase includes first iteration in which complete image of virtual machine is transferred. This causes total number of pages to be transferred always more than the virtual machine memory size. Iterations ‘2’ to ‘$n-1$’ come in second phase and all updated or dirty pages are transferred iteratively during this phase. As a result, same pages may be transferred repeatedly. The last phase includes stop and copy or *n*th iteration in which all remaining pages are transferred. After this, virtual machine is stopped at source and resumed at target host and migration method is completed. There are two stopping conditions, which decide when iterative memory transfer phase is to be stopped and third phase is to be activated. First one is the total number of iterations reached to 29 which is default value and another is the dirty pages in previous iteration are less than or equal to 256 KB. Based on either of these two conditions, last iteration or third phase is activated with transfer of remaining pages and after that migration method is stopped.

Method presented in this paper works in all three phases and tries to reduce extra pages transferred during migration from all three phases. It effectively improves migration performance with less overhead.

### Performance parameters

Performance of migration method is measured through following four parameters.

Total Pages Transferred: It is the total number of pages transferred during migration. For better performance this value should be less and ideally, it should be equal to the total number of pages of virtual machine. But, in precopy method, it is always more due to iterative transfer of updated pages. The total pages transferred $V_{mig}$ is defined as the total number of pages in all *n* iterations.

$$\begin{aligned} V_{mig} = \sum \limits _{i=0}^n V_{i} \end{aligned}$$

where, $\hbox {V}_{i}$ is the number of pages transferred in *i*th iteration and *n* is the total number of iterations.

Total Migration Time: It is the time taken in complete transfer of virtual machine from source to target. It should be minimum for fast completion of migration. Due to the transfer of extra pages other than the virtual machine memory size, migration time is also increased in precopy method. It is defined as time taken by all *n* iterations during migration.

$$\begin{aligned} T_{mig} = \sum \limits _{i=0}^n T_{i} \end{aligned}$$

where, $\hbox {T}_{i}$ is the time taken by *i*th iteration.

Downtime: It is the time taken by migration process to stop virtual machine at source and resume at target host. It directly affects the service availability. This value depends on the remaining pages in the last iteration. Downtime is measured as the time taken by last iteration of migration process.

Overhead: It is the additional data transferred during migration which is defined as the redundancy ratio of total pages or data transferred to the actual pages or size of virtual machine.

$$\begin{aligned} R_{d} = \frac{V_{mig}}{V_{mem}} \end{aligned}$$

where, $R_{d}$ is redundancy ratio, $V_{mig}$ is total data transferred during migration, and $V_{mem}$ virtual memory size. With the increase in redundancy ratio, more overhead is imposed by the migration method and for better performance, redundancy ratio needs to be less.

It may also affect the performance of virtual machine or application running within it. Extra CPU or space usage, page dirty rate, network bandwidth consumptions, and total number of iterations are some other parameters which also effects migration and can be considered for improving the performance of migration method.

### Xen architecture

**Fig. 3:**
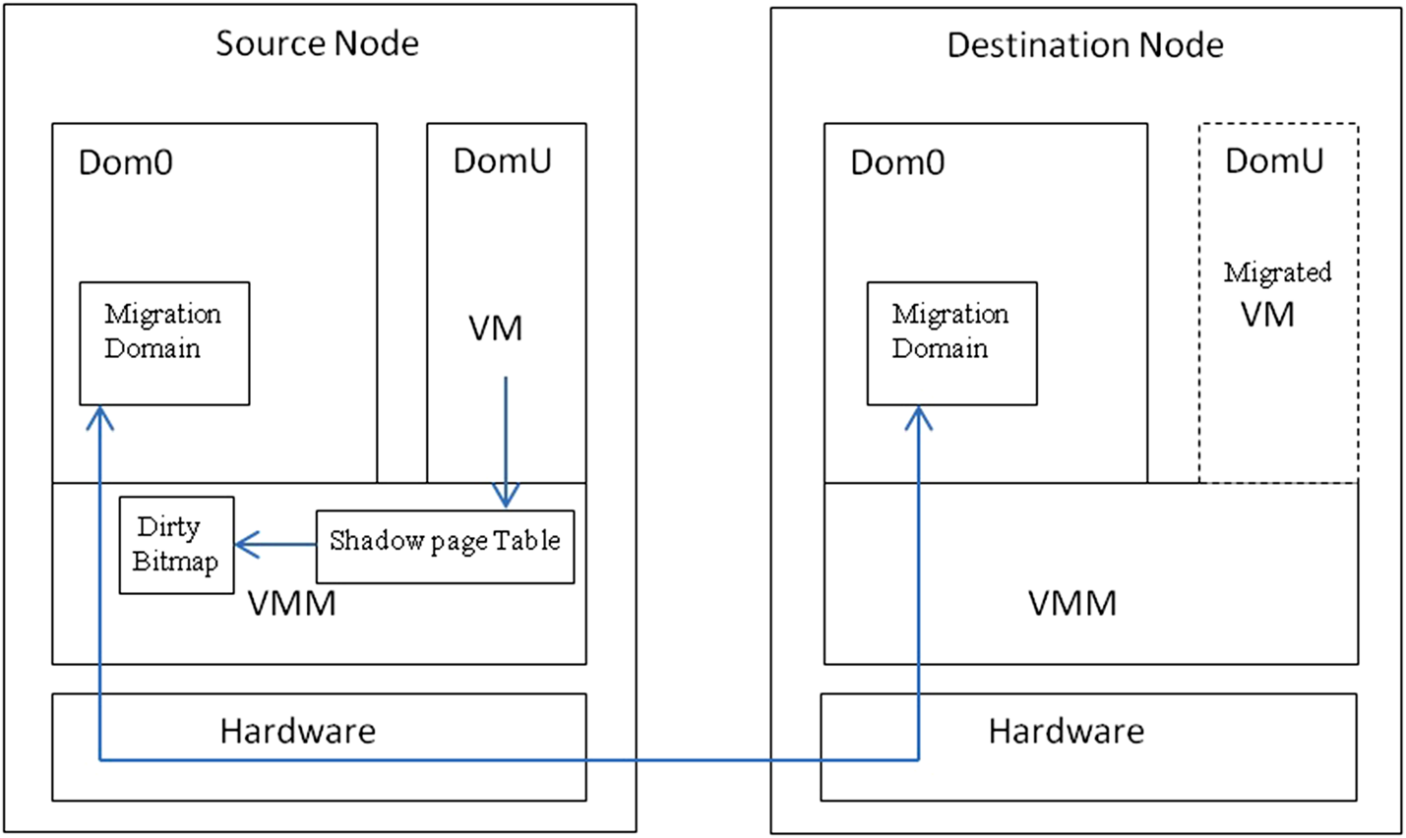
System structure of xen precopy method of VM live migration

Xen is a widely adopted as open source virtual machine monitor (VMM) or hypervisor. It supports virtualization and acts as a manager to run multiple operating systems on a common hardware by managing resources without affecting the performance. It also supports virtual machine live migration precopy method. For better understanding the mechanism of Xen virtual machine live migration, its important components are described. Figure 3 shows the basic system structure of Xen precopy method for virtual machine live migration. Xen hypervisor, domain 0 (Dom0) guest and Domain U guest are basic components of *Xen* virtual environment to support virtual machine live migration.

Xen hypervisor acts as a basic abstraction layer for guest operating system and hardware. It controls execution of virtual machine in common sharing environment. It also performs the task of CPU scheduling and memory partitioning of various virtual machines running on same hardware.

Domain 0 guest is a unique virtual machine with modified linux kernel running on a Xen hypervisor. It is a special privileged user, which has rights to access physical I/O resources and interact with other virtual machine too. For Xen virtualization environment, it is mandatory for domain 0 to run before any other virtual machine starts running. Domain management and control module is represented as the series of linux daemons, which support overall control and management of the virtualization environment. It resides within the domain 0 virtual machine.

Xend, Xm and Libxenctrl are the submodules of domain management and control module. Xend is a python application, which is considered as a manager for Xen environment. It handles the request coming from Xen hypervisor via Domain 0. Xm is a command line tool which takes input and delivers it to Xen via XML RPC (Remote Procedure call). Libxenctrl is a C library, that supports Xend to talk with Xen hypervisor via Domain 0. Domain U guest is unprivileged guest user which does not have direct access to physical hardware. It is managed through the Domain 0 guest.

Other than this, for the execution of precopy method, Xen uses some data structure for effective transfer of virtual machine memory pages during virtual machine live migration. Guest page table are managed by guest itself and pointed by CR3 register. Initially, Xen makes guest page tables read-only and when the guest tries to change or update its page table during migration, a page fault occurs. Xen employs shadow page table under the running virtual machine to log information of updated pages. It uses log of updated pages during migration. Xen also uses another bitmap named dirty log bitmap, which also contains the log of updated or dirty pages. When pages are updated during migration, the changes are propagated to both shadow page table and dirty log bitmap. Both are used to manage transferring of virtual machine pages at the time of migration and for each iteration bitmap is scanned to locate updated pages for transferring.

Xen itself optimizes precopy method by considering updated pages as writable working set (WWS) for each round. Pages within WWS are sent at last iteration. For this, it uses three types of bitmap named TO_SEND, TO_SKIP, and TO_FIX for tracking updated pages and to decide which pages are transferred in the current iteration.

TO_SEND bitmap contains those pages which are to be transferred in next iteration. TO_SKIP bitmap contains those pages which are skipped from being transferred in current iteration. These are the pages which are considered as frequently updated pages within WWS. TO_FIX bitmap contains those pages which are transferred at last iteration. By using these three bitmaps at each iteration during migration; precopy method decides which pages are transferred in a current iteration. Barham et al. (2003) presents Xen and its architecture in detail and effectively.

## Three phase optimization (TPO) method

In order to overcome the overhead of extra memory pages transfer in each iteration of precopy method, designing of such an algorithm is essential which can optimize the migration method at each iteration. This paper proposes a method, named as TPO, which minimizes number of memory pages to be transferred in each iteration of the migration method.

### Method design

The proposed method is implemented through three phases:

- First Phase: This phase deals with transferring of memory pages in first iteration of the migration method.
- Second Phase: In this phase, page transferring between iteration ‘2’ to ‘$\hbox {n}-1$’ is considered.
- Third Phase: Last iteration takes place under this phase.

**Fig. 4:**
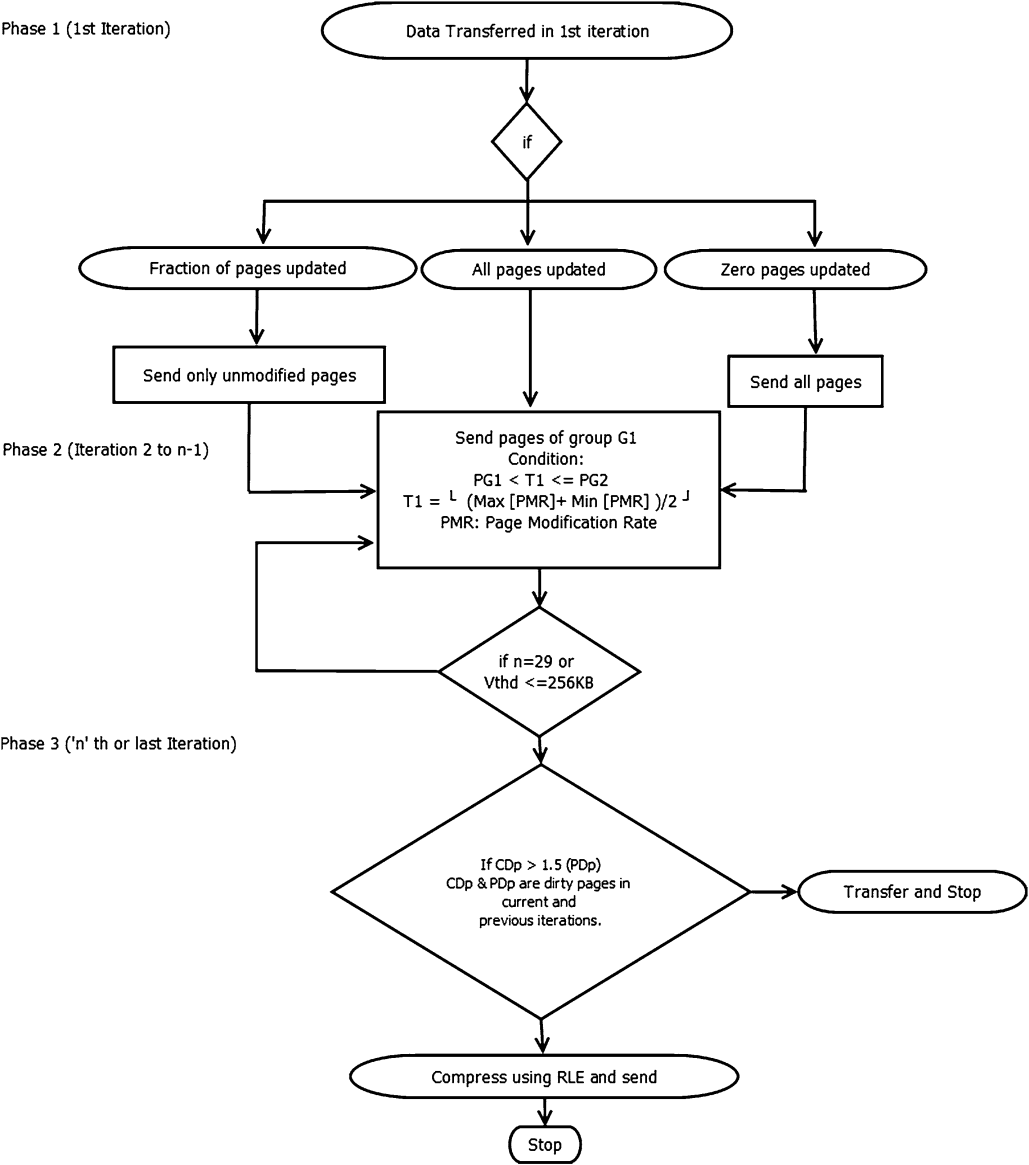
Flowchart of TPO method

Flow chart of the TPO method is shown in Fig. 4. All three phases are discussed in detail as follows:

**First Phase:** This phase covers transferring of memory pages in the first iteration. Transfer of virtual machine memory pages in the first phase of migration method is based on following three conditions:

1. If none of the memory pages is modified/updated (which is practically a rare case) then all the memory pages are transferred like precopy method.
2. If all memory pages are modified/updated (which is also a rare case) then those pages are transferred which are marked by the historical statistics based prediction method explained below in phase two.
3. If fraction of memory pages are modified/updated then only unmodified pages are transferred.

Since, most of the pages are modified in high load or write intensive workload, less number of pages is remain unmodified and hence, number of transferred pages reduces in first phase. This effectively reduces the total number of pages transferred during the migration method.

**Second Phase:** In this phase from iteration ‘2 to $\hbox {n}-1$’, iterative transfer of updated or dirty pages is reduced by using the historical statistics of updated pages to predict or trace frequently updated pages (WWS). The correct prediction or tracing of frequently updated pages reduce the transfer of same pages repeatedly during iterative transfer phase. As a result, transfer of similar pages multiple times during migration is reduced. To count the number of times a page can be updated, the virtual machine memory write functionality of hypervisor is modified. TO_SEND_h [i] array is used to store the count, which shows the number of times a page has been updated. Two byte space is used to store each entry in TO_SEND_h [i] array. By using the TO_SEND_h [i] array updated pages are divided into two groups $G_{1}$ and $G_{2}$ and the condition is

$$\begin{aligned} P_{G_{1}} < T_{1} \le P_{G_{2}} \end{aligned}$$

Pages updated less than a threshold value $T_{1}$ are considered as less frequently updated pages and kept in group $G_{1}$, whereas pages updated more than or equal to the threshold value $T_{1}$ are considered as frequently updated pages and kept in group $G_{2}$. At each iteration pages of $G_{1}$ (less frequently updated pages) group is transferred. This idea of grouping is inspired from the clustering method in which similar elements having same characteristics are combined into one group. Each time it gives more accurate and tighter bounded WWS window of frequently updated pages. Here, value of threshold is not static; it is updated at each iteration as following formula:

$$\begin{aligned} T_{1} = \lfloor [(\max [page\ modification\ rate] + \min [page\ modification\ rate]) \div 2] \rfloor \end{aligned}$$

In each iteration $T_{1}$ is calculated by using TO_SEND_h[i] array. Keeping the frequently updated pages till last iteration reduces the unnecessary transfer of same pages repeatedly and reduces total pages transferred as well as the migration time.

**Third Phase:** This phase is termed as stop and copy phase. In this phase, remaining pages are transferred based on two stopping conditions. The first stopping condition is the same as the stopping condition in precopy method. The second stopping condition states that if the number of dirty pages in last iteration is larger than 1.5 times of the number of dirty pages in the previous iteration, the pages are transferred after compression. The compression method used is Run Length encoding. This condition is employed to ensure that the downtime should not be unbearable.

Algorithm 1 shows the performance calculation of virtual machine live migration on different parameters like total pages transferred, total migration time and downtime, also given by (2011b).

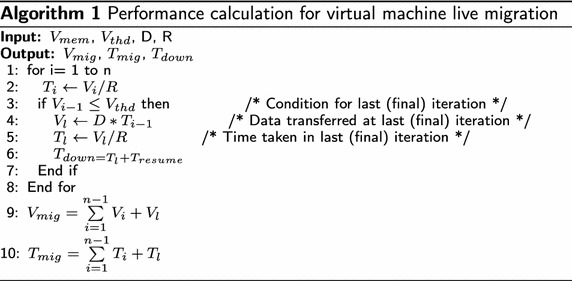



Algorithm calculates the time taken for transferring pages in each iteration. Total migration time or $T_{mig}$ is calculated as the summation of time taken by all iteration for transferring the complete virtual machine. Similarly, total pages transferred or $V_{mig}$ is calculated as the summation of pages transferred in all iterations. $T_{l}$ gives the value of downtime, which is the time taken in transferring pages in last iteration. Condition which is needed to be satisfied for executing the last iteration is, that the number of iterations reaches to its maximum value or the dirty pages in previous iteration are less then or equal to $V_{thd}$. The key parameters with their notations used in algorithm for performance calculation of virtual machine live migration are shown in Table 1.

**Table 1:** Performance parameters used in virtual machine live migration method

$V_{mem}$	Memory size of VM
$T_{mig}$	Total migration time
$V_{mig}$	Total pages transferred
$T_{down}$	Downtime
R	Memory transmission rate
D	Memory dirty rate per iteration
$V_{i}$	Volume of pages transferred at *i*th iteration
$T_{i}$	Time taken for transferring pages at *i*th iteration
$V_{l}$	Volume of Pages transferred in last iteration
$T_{l}$	Time taken for transferring pages in last iteration
$V_{thd}$	Threshold value required for last iteration
$T_{resume}$	Time taken by VM to resume at target
n	Maximum number of iterations
Temp array used	TO_SEND_h to store historical statistics based on number of times page is updated

## Simulation and performance evaluation

This section explains the simulation environment which evaluates the performance of the TPO method. It first presents simulation setup on which experiments are performed along with virtual machine workloads. It shows the evaluation of performance parameters viz. total pages transferred, total migration time, and downtime. It also presents the overhead analysis for the TPO method. To validate the effectiveness of TPO method, it is compared with precopy method as well as with other counterpart works. It also presents results which show the effectiveness of TPO method with less number of iterations.

### Simulation setup

Virtualization environment has been created using the following simulation setup: 2GB RAM, Intel Core i5 2400 CPU @ 3.10 GHz processor and VT-X technology enabled. The host machines were installed with CentOS 6.3 as the operating system with the hypervisor Xen 4.3.0. Virtual machines are also installed with Centos 6.3 as guest operating systems and configured with 2 VCPU and 1GB RAM. For migration virtual machine storage is exported as a system image file and accessed with NFS protocol. Number of seeds are ten and results are taken over average of all seeds. The performance of the method is tested under following four standard workloads:

1. Idle: An idle booted Centos operating system without any running application.
2. Kernel-built: Compiled a kernel source in the virtual machine to represent the system-call intensive workload.
3. Memtester 4.3: It is a utility program for testing memory subsystems for faults. It is used to impose memory workload.
4. Stress 1.0.1: It is a tool to impose a configurable amount of CPU, memory, I/O and disk stress. It is used to generate higher workload.

All the statistics like the number of pages sent, number of iterations, time taken for each iteration are extracted from log file generated during migration through Xen. The four workloads are executed with both precopy and the TPO for comparison. The performance of TPO is compared with basic precopy method corresponding to all three parameters viz. Total pages transferred, Total migration time and Downtime. The results of TPO method are also compared with reuse-distance (Alamdari and Zamanifar 2012) method and with some other counterpart methods.

### Comparison with Precopy Method

**Fig. 5:**
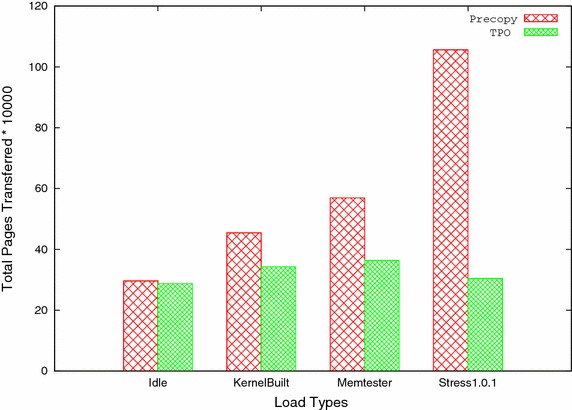
Total pages transferred versus load types

A. Total Pages Transferred: It shows the number of pages transferred during migration. Table 2 and Fig. 5 show the total pages transferred in both precopy and TPO for all four workloads. It shows that TPO performs better than precopy by reducing number of pages transferred in all four workloads. When virtual machine is under low workload like idle case, less pages are updated and most of the pages sent only once. This is the reason that, total pages transferred in TPO method as compared to precopy method is reduced by 2.83 %. For the case of heavy work load or write-intensive load, many pages get updated and sent frequently in precopy method. Number of pages sent multiple times in precopy method is considerably reduced in TPO method because in the latter, most frequently updated pages are kept till last iteration and then sent. As a result, TPO performs well and significantly reduces total pages transferred.

**Table 2:** Total pages transferred

System status	Precopy	TPO	Reduction ratio (%)
Idle	296,354	287,978	2.83
Kernel compile	454,311	342,951	24.51
Memtester 4.3	569,590	363,906	36.11
Stress 1.0.1	1,056,144	304,664	71.15

**Fig. 6:**
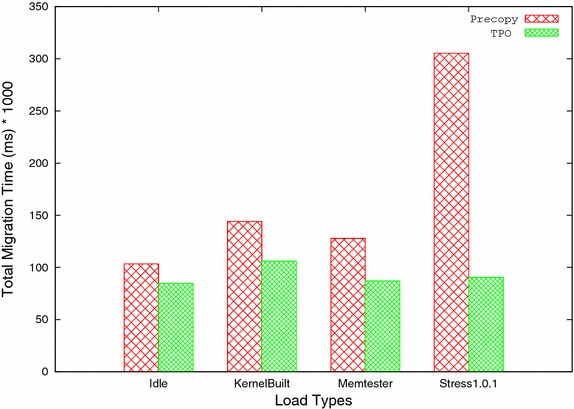
Total migration time versus load types

 B. Total Migration time: It is the time taken by all the ‘n’ iterations during migration. TPO effectively reduces the number of pages transferred in each iteration during migration by keeping frequently updated pages till last iteration. Due to this, time taken by all iterations is reduced, which results in reduction of total migration time. Table 3 and Fig. 6 show the total migration time taken by both precopy and TPO, which clearly shows that TPO performs well and reduces total migration time significantly for all four workloads.

**Table 3:** Total migration time

System status	Precopy	TPO	Reduction ratio (%)
Idle	103,435	84,871	17.95
Kernel compile	144,101	106,035	26.42
Memtester 4.3	127,802	87,152	31.74
Stress 1.0.1	305,277	90,434	70.38

**Table 4:** Downtime

System status	Precopy	TPO	Reduction ratio (%)
Idle	233	231	0.9
Kernel compile	6570	3521	46.4
Memtester 4.3	73,235	70,024	4.39
Stress 1.0.1	62,820	60,699	3.4

**Fig. 7:**
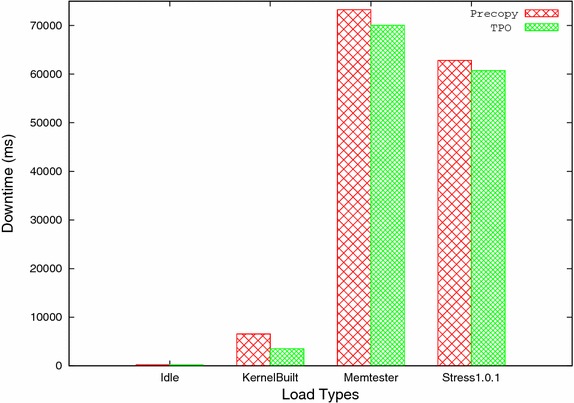
Downtime versus load types

C. Downtime: It is the time taken by the last stop and copy iteration, in which virtual machine is stopped at source host and resumed at target host after transferring remaining memory pages. It is one of the important parameter which directly affects the service availability. Its value should be as minimum as possible. Table 4 and Fig. 7 show the downtime for both methods with all four workloads. Downtime in TPO for idle case is almost equal to precopy method. TPO gives less downtime than precopy method for higher workload. It is due to reduction in transfer of extra pages in last iteration because only frequently updated pages are kept till last iteration while rest of the pages are transferred during previous iterations.

### Comparison with reuse-distance (Alamdari and Zamanifar 2012) method

The TPO is also compared with prediction based reuse-distance (Alamdari and Zamanifar 2012) method for the same sort of parameters and workloads. Tables 5, 6, and 7 show the difference in reduction ratio between them for each performance parameter viz. total page transferred, total migration time and downtime. Both methods are compared with their reduction ratio corresponding to precopy method. From the results, it is clear that TPO also improves all the performance parameters in comparison to reuse-distance method.

**Table 5:** Total pages transferred

System status	Reuse-distance (Alamdari and Zamanifar 2012) compared with precopy (%)	TPO compared with precopy (%)	Difference in reduction ratio (%)
Idle	0.27	2.83	2.56
Kernel compile	18.1	24.51	6.4
Memtester 4.3	31.9	36.11	4.21
Stress 1.0.1	68	71.15	3.15

**Table 6:** Total migration time

System status	Reuse-distance (Alamdari and Zamanifar 2012) compared with precopy (%)	TPO compared with precopy (%)	Difference in reduction ratio (%)
Idle	16.5	17.95	1.4
Kernel compile	22.2	26.42	4.2
Memtester 4.3	22.8	31.74	8.94
Stress 1.0.1	62.3	70.38	8.08

**Table 7:** Downtime

System status	Reuse-distance (Alamdari and Zamanifar 2012) compared with precopy (%)	TPO compared with precopy (%)	Difference in reduction ratio (%)
Idle	$-53$	0.9	53.9
Kernel compile	40.1	46.4	6.3
Memtester 4.3	1.8	4.39	2.59
Stress 1.0.1	0.4	3.4	3

TPO method performs well as compared to reuse-distance method because it only transfers unmodified pages in first iteration and less frequently updated pages in further iterations. In TPO method, the number of pages transferred is minimized effectively. Whereas, reuse-distance method sends all memory pages in first iteration, which is big in number and greatly increases the total number of pages transferred.

### Overhead analysis

**Fig. 8:**
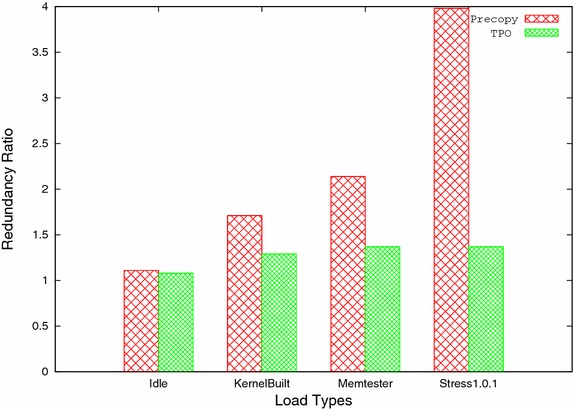
Redundancy ratio

Figure 8 shows the redundancy ratio for both precopy and TPO with all four workloads. From the figure it is clear that the overhead induced by the TPO method is significantly less as compared to precopy method. Less overhead makes TPO method more efficient in terms of bandwidth utilization and migration time. Other than this, TPO is also analyzed for space and processing overheads. The TPO uses TO_SEND_h[i] array to store the count for each memory page, based on number of times it is updated. Majority of times, it will have less entries than total number of pages and in worst case, it will be equal to the total number of pages of virtual machine. In worst case, space overhead 0.05% (for page size 4KB) is used to store the TO_SEND_h[i] array, which is very less and easily bearable. All the processing (prediction of frequently updated pages) are performed on the host machine, so there is no impact of processing on virtual machine. Memory allocation and de-allocation is dynamic in nature and hence, automatically freed after virtual machine migration. Similar bitmaps have also been used in other works (Svärd et al. 2011; Hu et al. 2011; Ma et al. 2010).

### Comparison with other methods

**Table 8:** Comparison of various migration methods along with TPO method

S.no.	Migration techniques	References	Parameters			Overhead	Workload
			TPT	TMT	DT		
1.	Compression based	Ma et al. (2012)	50.5 % $\downarrow$	48.2 % $\downarrow$	47.6 % $\downarrow$	—	Idle, static web, dynamic web, stream video, kernel compiler
		Svärd et al. (2011)	51 % $\downarrow$	35 % $\downarrow$	Factor 10–20 $\downarrow$	—	LMBench benchmark, streaming video server
		Zhang et al. (2010)	56.6 % $\downarrow$	34.9 % $\downarrow$	23.16 % $\downarrow$	—	Compilation, VOD, static web, dynamic web
		Jin et al. (2009)	68.8 % $\downarrow$	32 % $\downarrow$	27.1 % $\downarrow$	—	Static web, dynamic web, kernel-compiler, dbench, MUMmer (memory-intensive)
2.	CPU scheduling based	Jin et al. (2011)	—	1s $\uparrow$ in TMT	88 % $\downarrow$	6 % More CPU usage	Static web, dynamic web
3.	Shared storage based	Jo et al. (2013)	—	30 % $\downarrow$	—	0.2 % Space	RDesk, admin, file I/O
4.	Prediction of frequently updated pages based	Alamdari and Zamanifar (2012)	68 % $\downarrow$	62.3 % $\downarrow$	0.4 % $\downarrow$	—	Idle, kernel built, memtester 4.3 (memory-intensive), stress 1.0.1
		Ma et al. (2010)	34 % $\downarrow$	32.5 % $\downarrow$	$\uparrow$*	—	MUMmer (memory-intensive)
		Liu et al. (2011)	Reduced migration cost 72.9 % in terms of MT and DT*			—	Linux idle, TPC-C, Dbench, LINPACK, SPECweb2005
		Hu et al. (2011)	$\uparrow$* Bound no. of iterations 3–5	$\uparrow$*	$\uparrow$*	—	Low and high dirty page environment
		TPO	71.1 % $\downarrow$	70.4 % $\downarrow$	3.4 % $\downarrow$	0.05 % Space	Idle, kernel built, memtester 4.3 (memory-intensive), stress 1.0.1

Here, $\downarrow$ is decrease in value, $\uparrow$ is increase in value, ‘—’ shows parameter not specified, TPT is Total Pages Transferred, TMT is Total Migration Time, DT is Downtime, $\uparrow *$ is improvement but with no data specified, MT is Migration time, DT* is Data Transferred

TPO is also compared with other works for analyzing it more noticeably. Table 8 gives a comparative analysis for the behavior of TPO and other methods as compared to precopy corresponding to the parameters such as total page transferred, total migration time, downtime, overhead, experimental workloads used and their migration techniques used.

### Effect of number of iterations

**Fig. 9:**
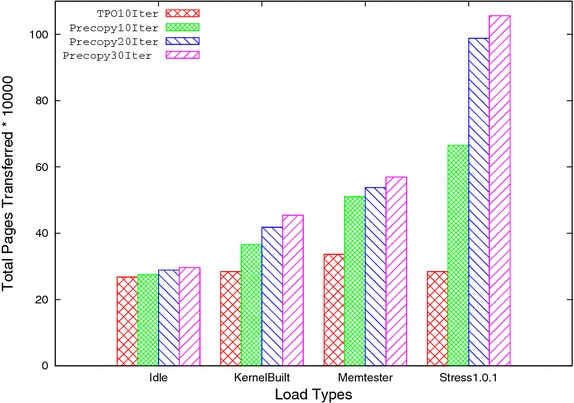
Total pages transferred

**Fig. 10:**
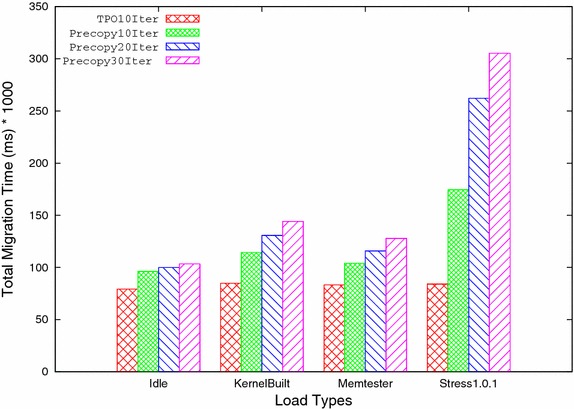
Total migration time

**Fig. 11:**
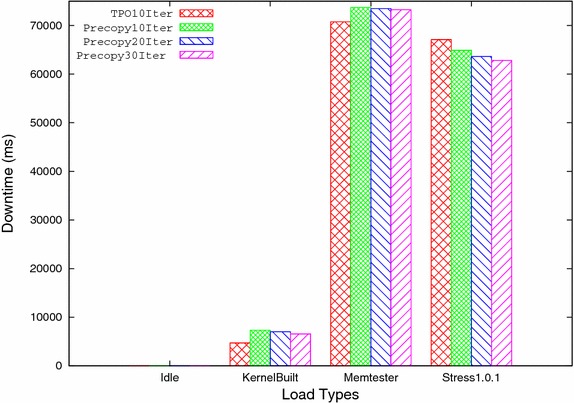
Downtime

Number of iterations is one of the important factors which affects the migration time. In order to analyze the effect of number of iterations, both TPO and precopy methods are run for 10, 20, and 30 iterations respectively and compared with each other. After analyzing both methods at different number of iterations, it is found that by reducing the numbers of iterations both total pages transferred, and total migration time are reduced considerably but downtime increases by a small amount. The analysis also shows that the TPO further reduces total pages transferred, and total migration time for 10 iterations, for all types of workloads because the transfer of redundant pages in subsequent iterations is reduced. Downtime is also reduced for rest of workloads except for higher workload such as stress 1.0.1 where frequency of page updation is high. Table 9 shows the improvement of all three performance parameters viz. total pages transferred, total migration time and downtime (except for stress) by TPO for 10 iterations compared to 30 iterations of the precopy method at different workloads. Figures 9, 10, and 11 shows the comparison of TPO at iteration 10 with the precopy method at 10, 20, and 30 iterations respectively.

**Table 9:** Percentage improvement of TPO method for 10 iterations over precopy for 30 iterations

S.no.	Parameters	Idle	Kernel compile (%)	Memtester 4.3 (%)	Stress 1.0.1 (%)
1	Total pages transferred	9.6 %	37.45	40.93	73.12
2	Total migration time	23.3 %	41.05	34.85	72.46
3	Downtime	$\approx$ Equal	28.4	3.4	$-6.4$

From above analysis, it can be concluded that if number of iterations are chosen dynamically according to the workloads then the migration method performs more efficiently and gives better performance.

## Open issues

The virtualization technology provides the ability to transfer virtual machine from one physical host to another using virtual machine live/offline migration. In order to keep transfer time and overhead minimum, efficient virtual machine live migration methods are required. Apart from this, other issues are also there which need to be addressed to make the migration method applicable on most of the virtual environments. This section identifies and discusses these key issues.

WAN (Wide Area Network) Environment: Most of the migration methods consider LAN (Local area network) environment for the migration, in which they have fair amount of available network bandwidth for the transfer of memory pages. But, the scenario will change if it moves towards the WAN. In the WAN, virtual machines are moved over the geographically separated cloud locations, having high latency and low network bandwidth as compared to LAN. Migration of big virtual machines over WAN with low service delay is very challenging and needs different solutions and techniques unlike traditional method used for LAN environment.

Bose et al. (2011) presents an optimized virtual machine live migration method over WAN environment. It replicates the copy of virtual machine at different sites. Scheduler selects the primary copy for updating, based on changing cost parameters, which gives the cost associated with the selection of primary copy for updation and also propagates the changes to other copies. This method is highly dependent on the replica placement strategies and on the ration of additional storage requirement. Hirofuchi et al. (2009) also uses two key techniques viz., on-demand fetching and background copying for the efficient virtual machine migration over WAN.

Liu et al. (2015) presents the VMbuddies coordination system to correlate virtual machine migrations within a WAN environment. By using synchronize protocol and an optimal network bandwidth allocation method, VMbuddies coordinates virtual machine migrations to minimize the migration cost. It also effectively reduces the performance degradation. In this method, the virtual machines disk image is replicated before the memory migration starts in WAN environment.

Concurrent Migration of Multiple Virtual Machines: Transfer of more than one virtual machines concurrently in a virtualized environment leads to increase in performance overhead and complexity. To maintain the efficiency of virtual machine live migration along with overhead and complexity, improvement in traditional virtual machine live migration method is required.

Energy Consumption: In present scenario, the energy consumption and carbon emission by the datacenter is a serious issue. When virtual machines are migrated within datacenter, additional energy is consumed by it which impacts on the carbon emitted by the datacenter. This issue is discussed by Beloglazov et al. (2012), in which it presents the energy-aware resource allocation method for allocating resources within data centers and shows that the migration also increases energy consumption. This concludes the need of energy efficient live migration methods.

Security: In the rush to take advantage of the virtual machine live migration, security issues are often ignored. During migration, virtual machine is moved over an insecure channel and stored on some another machine. It is the point where the intruder can get control over the virtual machine and simultaneously affect the target machine. Another issue is virtual machine authentication i.e., only legitimate user should be enabling the virtual machine migration. These security issues are needed to be handled during virtual machine live migration. Zhang et al. (2008) discusses the virtual machine migration security issues and proposes a secure migration system. A detail survey on cloud security is discussed by Vaquero et al. (2011), which helps to better understand the security as a challenge.

## Conclusion and future work

Virtual machine live migration is a process of moving virtual machine across hosts within a virtualized datacenter. Large number of virtual machine migration consumes high amount of network bandwidth as well as imposes performance overhead which adversely affects the overall efficiency of the system. As a solution, efficient migration methods are required which improve the migration process as well as impose minimum overhead. A virtual machine live migration method is evaluated based on the performance parameters such as number of pages transferred during migration, time to complete the migration process and downtime. Precopy method is one of the popular virtual machine live migration method, which iteratively transfers updated memory pages to migrate consistent image of virtual machine. Various work has been proposed to make precopy method more efficient by optimizing above parameters. This paper explores these works in detail. The main drawback of precopy method is transferring of all updated pages in each iteration which results into multiple time transfer of same pages.

In order to solve this problem, this paper proposes three phase optimization (TPO) method, which divides the whole migration process into three phases. The main aim of classifying the iterations into different phases is to minimize the transferring of redundant memory pages by applying different techniques in different phases. First phase consist of iteration one, in which only unmodified pages are transferred instead of complete virtual machine image. Second phase consists of iterations 2 to ‘$\hbox {n}-1$’, in which less frequently updated pages are sent. For this, TPO method classifies pages into less updated and frequently updated pages with the help of historical statistics of memory pages updated. It is inspired from the clustering method which classifies the elements of same property in one group. Third phase covers last iteration of migration method, which limits the downtime by using RLE compression method, only when number of remaining pages are very high.

The experimental results show that the TPO method effectively improves the migration performance by improving the total pages transferred by 71 %, total migration time by 70 % and downtime by 3 % with respect to the precopy method for higher workload. This paper also shows the analysis of precopy and TPO methods for different number of iterations. Proposed TPO method for 10 iterations is compared with precopy for 30 iterations on all types of workload. It improves total pages transferred by 73 %, and total migration time by 72 %, with little increasing downtime for higher workloads where page updating rate is very high. From this analysis, it can be suggested that if the number of iterations is kept dynamic in place of static then the TPO further improves the performance of migration method. It will be studied in more detail in future.

This paper also identifies and discusses some of the open issues related to virtual machine live migration and aimed to work on these issues in order to increase the applicability of virtual machine live migration method. In future, the research work is planned to migrate multiple virtual machines concurrently along with maintaining the performance of migration method with minimum overhead in terms of space and processing. Making TPO method energy efficient is also considered in future plan. Further, it is also planned to make proposed TPO method eligible to run over WAN environment which spreads virtualization across large geographical area.

## References

[CR1] Alamdari JF, Zamanifar K (2012) A reuse distance based precopy approach to improve live migration of virtual machines. In: 2012 2nd IEEE international conference on parallel distributed and grid computing (PDGC), IEEE, pp 551–556

[CR2] Barham P, Dragovic B, Fraser K, Hand S, Harris T, Ho A, Neugebauer R, Pratt I, Warfield A (2003). Xen and the art of virtualization. ACM SIGOPS Oper Syst Rev.

[CR3] Beloglazov A, Abawajy J, Buyya R (2012). Energy-aware resource allocation heuristics for efficient management of data centers for cloud computing. Future Gener Comput Syst.

[CR4] Bose SK, Brock S, Skeoch R, Rao S (2011) Cloudspider: combining replication with scheduling for optimizing live migration of virtual machines across wide area networks. In: 2011 11th IEEE/ACM international symposium on cluster, cloud and grid computing (CCGrid), IEEE, pp 13–22

[CR5] Clark C, Fraser K, Hand S, Hansen JG, Jul E, Limpach C, Pratt I, Warfield A (2005) Live migration of virtual machines. In: Proceedings of the 2nd conference on symposium on networked systems design & implementation-volume 2. USENIX Association, pp 273–286

[CR6] Hirofuchi T, Ogawa H, Nakada H, Itoh S, Sekiguchi S (2009) A live storage migration mechanism over wan for relocatable virtual machine services on clouds. In: Proceedings of the 2009 9th IEEE/ACM international symposium on cluster computing and the grid. IEEE computer society, pp 460–465

[CR7] Hu B, Lei Z, Lei Y, Xu D, Li J (2011) A time-series based precopy approach for live migration of virtual machines. In: 2011 IEEE 17th international conference on parallel and distributed systems (ICPADS), IEEE, pp 947–952

[CR8] Jin H, Deng L, Wu S, Shi X, Pan X (2009) Live virtual machine migration with adaptive, memory compression. In: IEEE International conference on cluster computing and workshops, 2009. CLUSTER’09. IEEE, pp 1–10

[CR9] Jin H, Gao W, Wu S, Shi X, Wu X, Zhou F (2011). Optimizing the live migration of virtual machine by cpu scheduling. J Netw Comput Appl.

[CR10] Jo C, Gustafsson E, Son J, Egger B (2013) Efficient live migration of virtual machines using shared storage. In: ACM SIGPLAN notices, vol 48, ACM, pp 41–50

[CR11] *KVM*. http://www.linux-kvm.org

[CR12] Liu H, He B (2015). Vmbuddies: coordinating live migration of multi-tier applications in cloud environments. IEEE Trans Parallel Distrib Syst.

[CR13] Liu H, Jin H, Liao X, Yu C, Xu C-Z (2011). Live virtual machine migration via asynchronous replication and state synchronization. IEEE Trans Parallel Distrib Syst.

[CR15] Liu W, Fan T (2011a) Live migration of virtual machine based on recovering system and cpu scheduling. In: Information technology and artificial intelligence conference (ITAIC), 2011 6th IEEE joint international, vol 1. IEEE, pp 303–307

[CR14] Liu H, Xu C-Z, Jin H, Gong J, Liao X (2011b) Performance and energy modeling for live migration of virtual machines. In: Proceedings of the 20th international symposium on High performance distributed computing. ACM, pp 171–182

[CR16] Liu Z, Qu W, Liu W, Li K (2010) Xen live migration with slowdown scheduling algorithm. In: 2010 International conference on parallel and distributed computing, applications and technologies (PDCAT). IEEE, pp 215–221

[CR17] Ma F, Liu F, Liu Z (2010) Live virtual machine migration based on improved pre-copy approach. In: 2010 IEEE international conference on software engineering and service sciences (ICSESS). IEEE, pp 230–233

[CR18] Ma Y, Wang H, Dong J, Li Y, Cheng S (2012) Me2: efficient live migration of virtual machine with memory exploration and encoding. In: 2012 IEEE international conference on cluster computing (CLUSTER). IEEE, pp 610–613

[CR19] *Microsoft Corporation*. http://www.microsoft.com/en-us/server-cloud/hyper-v-server/

[CR20] Mishra M, Das A, Kulkarni P, Sahoo A (2012). Dynamic resource management using virtual machine migrations. IEEE Commun Mag.

[CR21] Nelson M, Lim B-H, Hutchins G et al. (2005) Fast transparent migration for virtual machines. In: USENIX annual technical conference, general track, pp 391–394

[CR22] Sharma S, Chawla M (2013) A technical review for efficient virtual machine migration. In: 2013 International conference on cloud & ubiquitous computing & emerging technologies (CUBE). IEEE, pp 20–25

[CR23] Svärd P, Tordsson J, Hudzia B, Elmroth E (2011) High performance live migration through dynamic page transfer reordering and compression. In: 2011 IEEE 3rd international conference on cloud computing technology and science (CloudCom). IEEE, pp 542–548

[CR24] Vaquero LM, Rodero-Merino L, Morán D (2011). Locking the sky: a survey on iaas cloud security. Computing.

[CR25] *VMware Incorporation*. http://www.vmware.com

[CR26] Waldspurger CA (2002). Memory resource management in vmware esx server. ACM SIGOPS Oper Syst Rev.

[CR27] *Xen*. http://www.archive.xenproject.org/files/Marketing/HowDoesXenWork.pdf

[CR28] Zhang F, Huang Y, Wang H, Chen H, Zang B, (2008), Palm: security preserving vm live migration for systems with vmm-enforced protection. In: Trusted infrastructure technologies conference (2008) APTC’08. Third Asia-Pacific, IEEE, pp 9–18

[CR29] Zhang X, Huo Z, Ma J, Meng D (2010) Exploiting data deduplication to accelerate live virtual machine migration. In: 2010 IEEE international conference on cluster computing (CLUSTER). IEEE, pp 88–96

